# Prevalance and risk factors of congenital malaria in Osogbo, Southwestern Nigeria

**DOI:** 10.3205/000343

**Published:** 2025-07-15

**Authors:** Saheed A. Odediji, Surakat A. Olabanji, Abdullahi O. Olawuyi, Kazeem O. Amoo, Basirat O. Amoo-Adeboye, Monsuru A. Adeleke

**Affiliations:** 1 Parasitology and Vector Biology Unit, Department of Zoology, Osun State University, Osogbo, Nigeria; 2 Department of Medical Laboratory Science, Federal University of Health Science Ila-Orangun, Nigeria; 3 Obafemi Awolowo University Teaching Hospital, Ile-Ife, Nigeria

**Keywords:** congenital malaria, prevalence, risk factors, Osogbo, Nigeria

## Abstract

**Background:**

Congenital malaria, defined as the presence of malaria parasites in the peripheral smear of newborns within the first seven days of life, remains an underexplored aspect of malaria epidemiology in endemic regions like Nigeria. This study investigated the prevalence and associated risk factors of congenital malaria at the State Specialist Hospital (SSH), Osogbo, Southwestern Nigeria.

**Method:**

This cross-sectional study collected peripheral and cord blood samples from 77 newborns within the first 24 hours of delivery. We examined blood samples for malaria parasites using both light microscopy and malaria rapid diagnostic test (mRDT) kits. A structured research proforma was used to assess various risk factors, including sociodemographic details, gravidity, parity, retroviral status, and malaria prevention measures during pregnancy.

**Results:**

The overall prevalence of congenital malaria was 6.5%, with all infants exhibiting congenital malaria also demonstrating cord blood parasitaemia. Maternal malarial parasitaemia emerged as a strong predictive factor (p<0.05), while factors such as maternal retroviral status, intermittent prophylactic therapy, therapeutic antimalarial use, and bednet usage were not significantly associated with congenital malaria (p>0.05).

**Conclusion:**

Our study observed a low prevalence of congenital malaria in Osogbo and emphasizes the significance of maternal malarial parasitaemia in predicting the condition. However, there is need for additional multicenter studies to comprehensively determine the prevalence of congenital malaria in Osun State and Nigeria, providing a basis for targeted interventions and healthcare strategies.

## Introduction

Malaria remains a significant global health concern, particularly in tropical regions, where it is one of the leading causes of disease and death in many developing countries [1]. Africa, especially sub-Saharan Africa, bears a disproportionate burden of this disease, accounting for around 95% of global cases and 96% of deaths in 2020, with six highly malaria-endemic African nations, including Nigeria, responsible for over half of all malaria-related fatalities worldwide [1], [2], [3]. The COVID-19 pandemic may have further exacerbated the situation by disrupting healthcare systems [4], [5]. 

Malaria also has a devastating impact on pregnant women and newborns, with around 26% of pregnant women experiencing fever and severe anemia, and the prevalence being higher during the first and second trimesters, contributing significantly to healthcare expenditures [6], [7]. Malaria causes over 240 million cases and over 600,000 deaths annually, mostly among children under the age of five years [8]. It leads to adverse birth outcomes, such as low birth weight (8–14% of cases), preterm delivery (8–36%), and intrauterine growth restriction (13–70%), which are associated with increased infant mortality, including an estimated 200,000 stillbirths annually in sub-Saharan Africa due to Plasmodium falciparum infection [9], [10]. Additionally, malaria imposes substantial direct and indirect costs, with the direct costs of illness, treatment, and premature death estimated at over $12 billion per year [11], and a negative impact on economic growth and productivity in affected regions [12].

Malaria is a mosquito-borne infectious parasitic disease that is prevalent in tropical and subtropical areas, with sub-Saharan Africa being a region that is hyper-endemic for Plasmodium falciparum – the most virulent species that causes malaria in humans [13], [14]. Vertical transmission of malaria from the mother to the fetus through the placenta (congenital malaria) is a recognized mode of transmission worldwide [15], [16].

Congenital malaria is defined as malaria parasite demonstrated in the peripheral smear of a newborn from 24 hours to seven days of life [17]. In endemic areas where mothers have acquired considerable immunity to malaria, infection with Plasmodium falciparum during pregnancy does not always cause symptomatic illness. Its diagnosis is based upon detection of asexual forms of malaria parasites on a blood smear of the peripheral blood of the newborn, or later if there is no possibility of postpartum infection through infective mosquito bites [18], [19].

The true prevalence of congenital malaria has been a subject of wide discrepancies across different countries of the world, and even among countries with stable malaria transmission all through the year [20], [21]. This variation in diagnosis of congenital malaria has relegated its diagnosis in clinical practice, and its consequential deleterious effects may be more than anticipated. The lack of data regarding its diagnosis and associated risk factors may lead to jettisoning of congenital malaria as a disease of priority in newborns. This necessitates the need for more robust research into the burden of congenital malaria and its possible risk factors in Nigeria, in order to inform meaningful policies to control it.

## Method

### Study design and setting

This cross-sectional study was carried out at the State Specialist Hospital (SSH), Asubiaro, Osogbo, Osun State, Southwestern Nigeria, over a period of 6 months (September 2022 to February 2023). Osun State is located in the tropical vegetation zone of Southwestern Nigeria, with high malaria transmission all year round.

### Study population and sampling

The study population comprised consecutive newborn babies delivered at the Labour Ward of the SSH, Osogbo. Inclusion criteria were neonates with estimated gestational age above 34 weeks and whose mothers consented to participate. Exclusion criteria included neonates whose mothers declined consent, with gestational age less than 35 weeks, infants of diabetic mothers, and neonates with major congenital abnormalities.

### Sample size determination

The sample size of 77 newborns was calculated using the Leslie Fisher’s formula, considering a prevalence of 5.3% from a previous study (16), a 95% confidence level, and a 5% margin of error.

### Specimen collection and processing

Thick and thin blood films were obtained from the cord blood and peripheral blood of each neonate within 24 hours of birth. Cord blood was collected after cleaning the cord with 70% alcohol to avoid maternal blood contamination. Neonatal peripheral blood was obtained from a peripheral vein on the dorsum of the hand. Duplicate thick and thin blood films were prepared from both cord and peripheral blood samples.

The blood films were stained with Giemsa and examined for the presence of malaria parasites under a light microscope by an experienced medical laboratory scientist and the researcher. Malaria rapid diagnostic test (mRDT) kits were also used to detect malaria parasites in the blood samples.

### Data collection

A structured research proforma was used to obtain information on sociodemographic characteristics, maternal factors (age, parity, education, occupation, use of malaria prophylaxis, use of insecticide-treated nets, use of therapeutic antimalarials, retroviral status), and neonatal factors (birth weight, gestational age).

### Ethical considerations

The study was approved by the Health Research Ethics Committee of the Specialist Hospital, Osogbo (protocol number: HREC/27/04/2015/SSHO/784). Informed parental consent was obtained from the mother of each neonate.

### Statistical analysis

Data was analyzed using IBM SPSS Statistics version 27. Descriptive statistics, chi-square tests, and logistic regression were used as appropriate. A p-value <0.05 was considered statistically significant.

## Results

### Sociodemographic characteristics

A total of 77 mother-baby pairs were enrolled. The mean maternal age was 25.6±4.8 years, and the mean parity was 2.1±1.0. Most babies (60, 77.9%) were delivered at 37 weeks gestational age or above, and the mean birth weight was 2.9±0.4 kg. Regarding maternal education, 55.8% had secondary education, 41.6% had tertiary education, and 2.6% had only primary education. The majority of the mothers were artisans (51.9%), followed by business owners (22.1%) and civil servants (15.6%).

### Prevalence of congenital malaria

Five (5) out of the 77 newborns had malaria parasitemia in their peripheral blood, giving a prevalence of congenital malaria of 6.5%. All 5 infants with congenital malaria also demonstrated parasitemia in their cord blood samples.

### Factors for congenital malaria

Maternal malarial parasitaemia was a significant risk factor for congenital malaria (p<0.05). However, factors such as maternal retroviral status, intermittent prophylactic therapy, therapeutic antimalarial use, and bednet usage were not significantly associated with congenital malaria.

### Comparison of the prevalence of congenital malaria with cord blood malaria parasitaemia

Of the 77 cord blood samples analyzed, 7 (9.1%) tested positive for malaria parasites. All five infants with congenital malaria were in this group. The remaining two infants with positive cord blood did not have malaria parasites in their peripheral blood, indicating that in-utero transmission occurs via the placenta and that cord blood parasitaemia does not always result in congenital malaria (p<0.05).

Maternal malaria was a significant factor, with 100% of mothers of infants with congenital malaria testing positive, compared to 12.5% of mothers in the non-congenital malaria group. Cord blood rapid diagnostic tests (RDTs) correctly identified 4 out of 5 congenital malaria cases, a statistically significant finding compared to the non-congenital group where no cases were detected by RDT.

## Discussion

Table 1 (Tab. 1) reveals no statistically significant differences (p>0.05) in maternal age, parity, gestational age, birth weight, education level, or occupation between neonates with and without congenital malaria in Osogbo. This indicates that these specific sociodemographic and basic neonatal factors were not primary determinants of congenital malaria risk in this cohort. Our finding implies that congenital malaria transmission in this endemic setting may be driven more directly by biological factors, such as active maternal parasitaemia rather than the sociodemographic parameters examined. This aligns with evidence from other endemic regions where maternal infection status and placental factors supersede broader sociodemographic influences as key determinants of vertical transmission [18].

In this study, we observed a relatively low prevalence of congenital malaria (6.5%) at the State Specialist Hospital, Osogbo, Southwestern Nigeria (Table 2 (Tab. 2)). This finding is consistent with previous studies conducted in endemic areas, which have reported a low incidence of congenital malaria despite high maternal malaria prevalence [16], [22], [23], [24]. However, this contrasts with earlier reports of a high prevalence of congenital malaria in some parts of Nigeria, such as Ile-Ife [15], [25].

The observed low prevalence of congenital malaria may be attributed to the protective effect of transplacentally acquired maternal antibodies, which can delay the onset of symptoms in newborns up to 3–6 weeks after birth [26]. This suggests that the placenta may effectively act as a barrier, preventing the transfer of malaria parasites, and the presence of fetal hemoglobin (HbF) in the neonate/fetus may also prevent high parasitemia [18].

The relatively low prevalence of congenital malaria observed in this study is an important finding, as it suggests that the burden of congenital malaria may not be as high as previously believed in this region. This has implications for the prioritization of congenital malaria as a public health concern and the development of targeted interventions.

Maternal malarial parasitemia emerged as a significant risk factor for congenital malaria in this study, while factors such as maternal retroviral status, use of intermittent prophylactic therapy, therapeutic antimalarial use, and bednet usage were not significantly associated with congenital malaria (Table 3 (Tab. 3)). The finding that maternal malarial parasitemia is a strong predictive factor for congenital malaria is in line with the established understanding that congenital malaria is a consequence of maternal clinical attacks of malaria during pregnancy [26].

However, the lack of association between congenital malaria and factors such as maternal retroviral status, use of intermittent prophylactic therapy, therapeutic antimalarial use, and bednet usage (Table 4 (Tab. 4)), contrasts with previous studies that have identified these as potential risk factors [22], [27]. This may indicate the complex interplay of host, parasite, and environmental factors in the epidemiology of congenital malaria.

The significant association between maternal malarial parasitemia and congenital malaria observed in this study suggests that the presence of malaria parasites in the maternal circulation during pregnancy increases the likelihood of transplacental transmission to the fetus. This is similar to what was found in other studies where cord-blood malarial parasitaemia was studied along with congenital malaria (using neonatal peripheral blood smear), where similar prevalence were gotten for both [28]. Hence, cord blood malarial parasitaemia is a good predictor of congenital malaria with a p-value of <0.001. This has important clinical implications, as it underscores the need for effective prevention and control of maternal malaria, which could in turn help reduce the burden of congenital malaria in this region.

From this study, maternal retroviral disease did not increase the risk of congenital malaria in their babies using univariate logistic regression. This is in variance with another study done where maternal retroviral disease has been found as a risk factor for malaria in pregnancy [27]. However, the study [27] did not indicate if the women with human immunodeficiency virus (HIV) (in that study) were on highly active anti-retroviral therapy (HAART), and if they had reduced viral load. The women with HIV from our study were all on HAART, and were all virologically suppressed (i.e viral load <1,000 copies/mL of blood). So, our result is not surprising as the probable aetio-pathogenesis of increased risk of malaria in HIV positive pregnant women is the “double” acquired immunosuppression caused by pregnancy and the HIV virus, and since the viral loads of the HIV mothers in this study were well suppressed, the immunosuppression from HIV was theoretically absent.

The use of both light microscopy and of malaria rapid diagnostic test (mRDT) in this study provided a more comprehensive assessment of congenital malaria diagnosis. The agreement between the two diagnostic methods (Table 2 (Tab. 2)) observed in this study underscores the utility of mRDT in the diagnosis of congenital malaria, which is a valuable finding given the limited availability of skilled microscopy services or in the absence of regular electricity supplying many resource-limited settings [29], [30], [31].

It is recommended that a larger multicenter study with a longer follow-up period would provide more comprehensive data on the prevalence of congenital malaria in the region. This will provide a stronger evidence base for the development of targeted interventions and healthcare strategies to address the burden of congenital malaria in the country.

## Conclusion

This study reports a relatively low prevalence of congenital malaria (6.5%) in Osogbo, Southwestern Nigeria, with maternal malarial parasitemia emerging as a significant risk factor. The cord blood parasitemia is unsurprisingly very predictive of peripheral blood parasitemia in the newborn, with 100% concordance rate. This may offer a good alternative blood specimen in newborns with suspected congenital malaria, as the cord blood may be collected in lieu of pricking a newborn to obtain blood from venipuncture. Again, a large amount of blood from the umbilical cord may be collected for congenital malaria diagnosis, without the fear of “physician-induced anaemia” from multiple venepunctures in newborns.

The study also underscores the utility of mRDT in the diagnosis of congenital malaria. These results have important implications for the prioritization of congenital malaria as a public health concern and the development of targeted interventions.

## Recommendations

Future research should consider larger multicenter studies to provide a more comprehensive understanding of the prevalence and risk factors of congenital malaria in Osun State and other regions of Nigeria [18]. A longitudinal study design would allow for better determination of the temporal relationship between identified risk factors and the development of congenital malaria. This stronger evidence base would inform the development of targeted interventions and healthcare strategies to address the burden of congenital malaria in the country.

Babies born to mothers with positive blood smear for malaria parasite should be screened and followed up for congenital malaria. Thus, routine screening for congenital malaria should be incorporated as part of standard newborn care, particularly in high-transmission settings. This would facilitate early diagnosis and prompt management of congenital malaria, potentially improving health outcomes for affected infants.

Furthermore, efforts to strengthen malaria prevention and control strategies, including the use of intermittent preventive treatment and insecticide-treated bednets, should be prioritized to reduce the burden of maternal malaria and, consequently, congenital malaria [27]. Improving the coverage and adherence to these interventions could have a significant impact on the prevalence of congenital malaria in the region.

Finally, healthcare facilities should enhance their capacity to diagnose congenital malaria using both microscopy and rapid diagnostic tests. Improving diagnostic capabilities would ensure timely identification and management of congenital malaria cases, thereby reducing the associated morbidity and mortality.

## Notes

### Acknowledgement

We express our gratitude to the staff and management of the State Specialist Hospital, Osogbo, Osun State, Nigeria, for their cooperation and support during this research study. We also extend our appreciation to the study participants for their willingness to contribute to this important research endeavour.

### Authors’ contributions

- Conception/design of study: Odediji SA, Adeleke MA, Surakat OA
- Data acquisition: Odediji SA, Adeleke MA, Surakat OA
- Data analysis/interpretation: Odediji SA, Adeleke MA, Surakat OA
- Drafting manuscript: Olawuyi AO, Odediji SA
- Critical revision of manuscript: Adeleke MA, Surakat OA, Olawuyi AO, Odediji SA, Amoo QA, Amoo-Adeboye BO
- Final approval and accountability: Adeleke MA, Surakat OA, Olawuyi AO, Odediji SA, Amoo QA, Amoo-Adeboye BO
- Technical or material support: Adeleke MA, Surakat OA, Olawuyi AO, Odediji SA, Amoo QA, Amoo-Adeboye BO
- Supervision: Adeleke MA, Surakat OA

### Competing interests

The authors declare that they have no competing interests.

## Figures and Tables

**Table 1:**
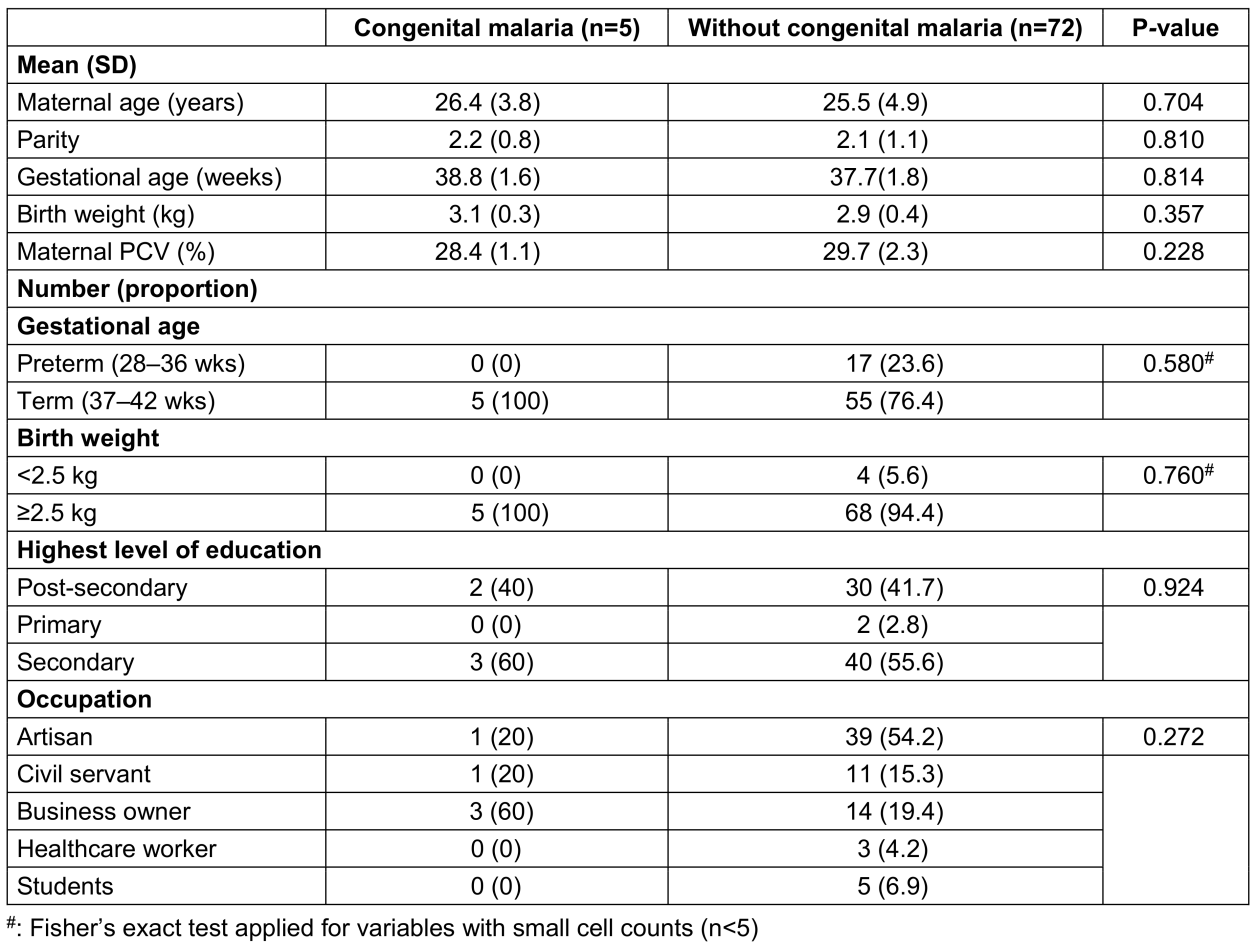
Sociodemographic characteristics mothers and newborn with and without congenital malaria

**Table 2:**
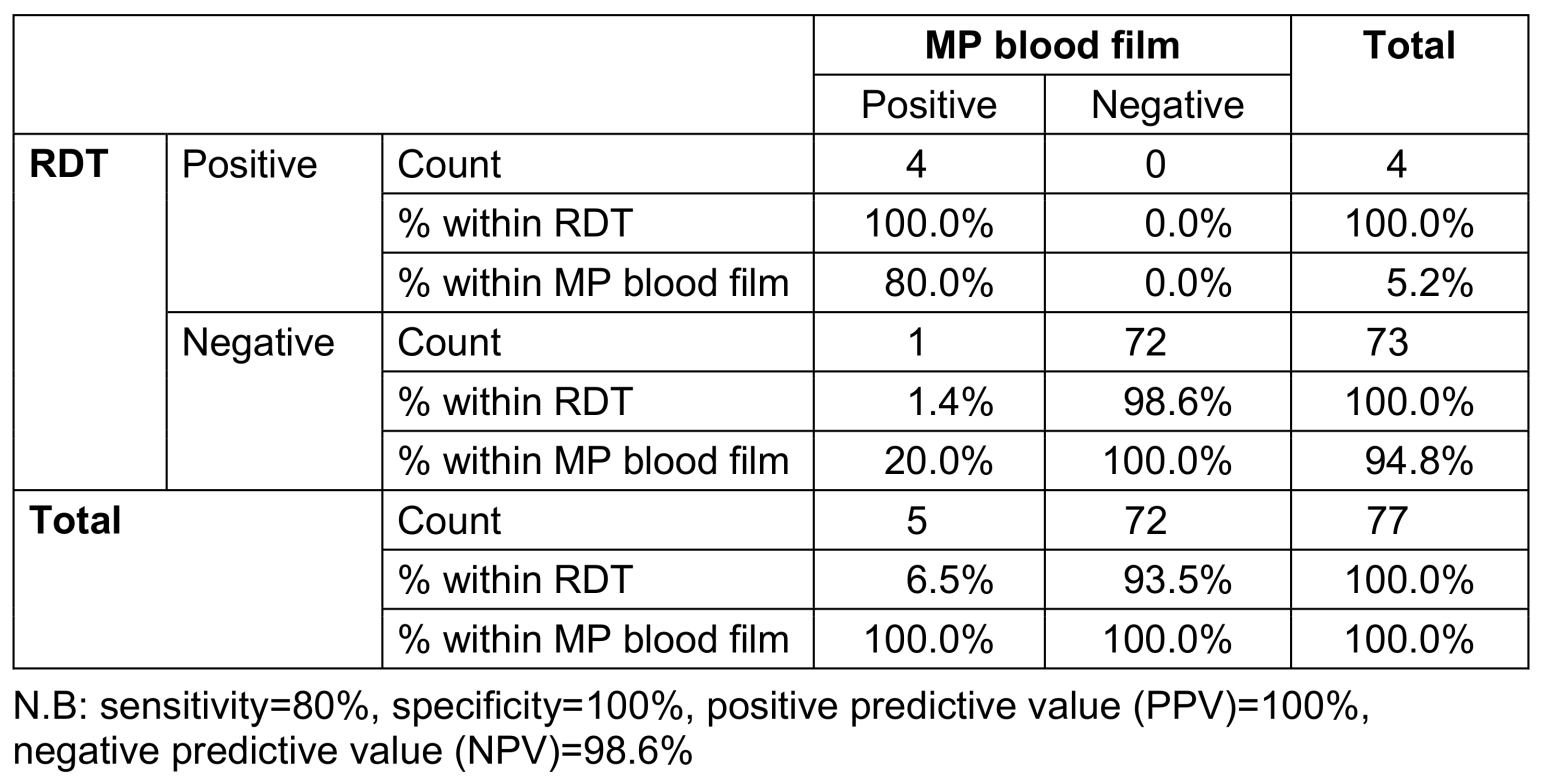
Diagnostic accuracy of malaria rapid diagnostic test (RDT) in relation to blood film

**Table 3:**
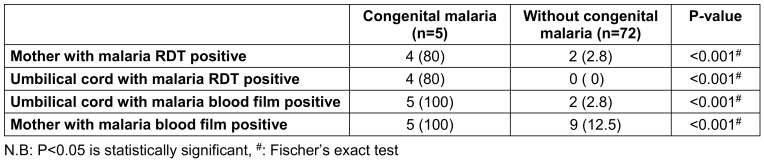
Results of maternal, cord blood malarial microscopy and rapid diagnostic tests

**Table 4:**
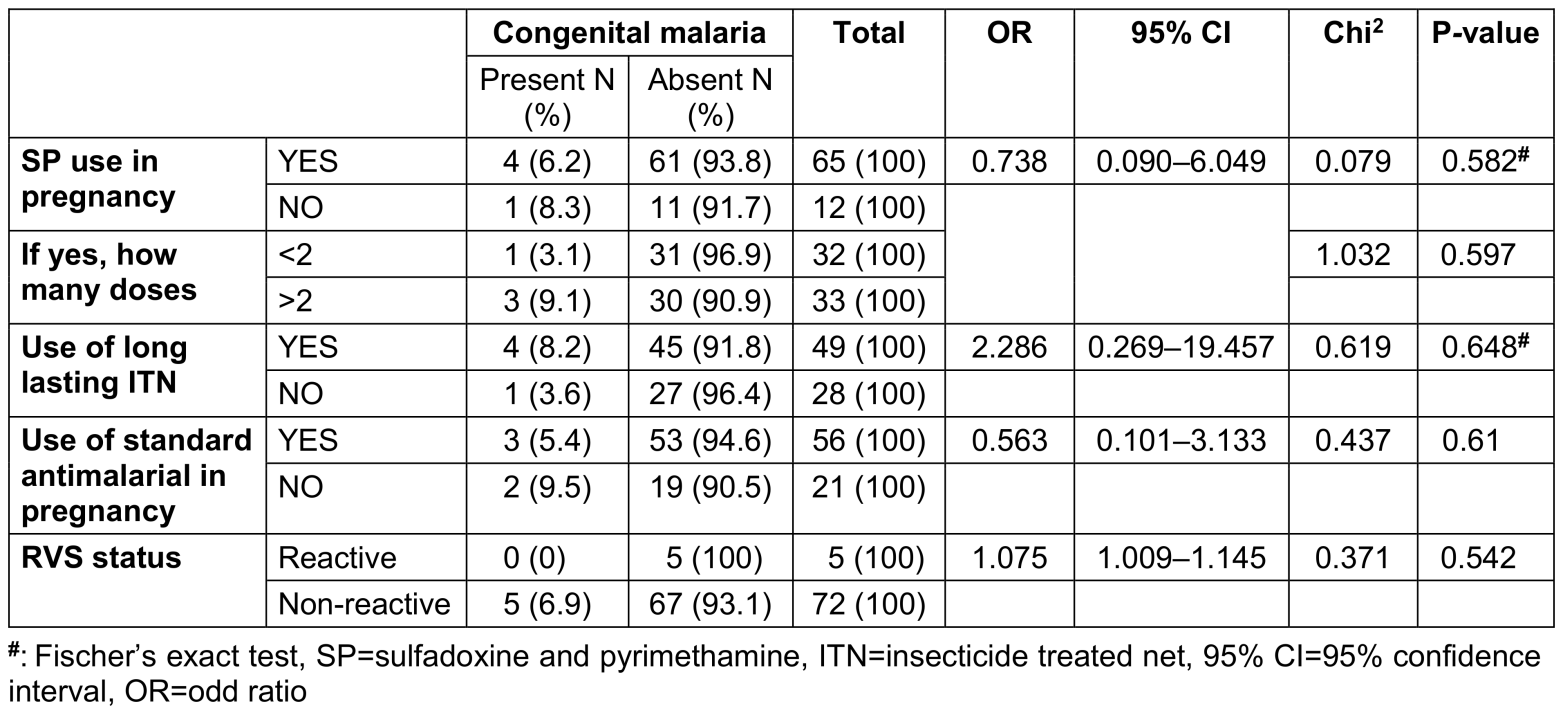
Risk factors of congenital malaria among mothers
